# Effect of Endoscopic Submucosal Dissection for Superficial Esophageal Neoplasms and Risk Factors for Postoperative Stricture

**DOI:** 10.1097/MD.0000000000000373

**Published:** 2015-01-09

**Authors:** Keita Funakawa, Hirofumi Uto, Fumisato Sasaki, Yuichiro Nasu, Seiichi Mawatari, Shiho Arima, Junichi Nakazawa, Hiroki Taguchi, Shinichi Hashimoto, Shuji Kanmura, Hitoshi Setoyama, Masatsugu Numata, Hirohito Tsubouchi, Akio Ido

**Affiliations:** From the Digestive and Lifestyle Diseases (KF, HU, FS, YN, SM, SA, JN, H. Taguchi, SH, SK, HS, MN, AI), Department of Human and Environmental Sciences, Kagoshima University Graduate School of Medical and Dental Sciences; Department of Internal Medicine (KF, H. Tsubouchi), Kagoshima City Hospital; and Department of HGF Tissue Repair and Regenerative Medicine (H. Tsubouchi, AI), Kagoshima University Graduate School of Medical and Dental Sciences, Kagoshima, Japan.

## Abstract

Endoscopic submucosal dissection (ESD) enables wider tumor resection compared with endoscopic mucosal resection and en bloc resection of superficial esophageal neoplasms. However, ESD may cause difficult-to-treat stricture of the esophagus, and therefore, prediction of and measures against postoperative esophageal stricture are critical. The aim of this study was to evaluate the effect of ESD on superficial esophageal neoplasms and identify risk factors associated with esophageal stricture after ESD.

This study included 165 lesions in 120 patients with superficial esophageal neoplasms, including cancer and neoplasia, who underwent ESD from 2009 to 2013.

The complete resection rate of superficial esophageal neoplasms by ESD was 90.9%. After ESD, 22 subjects (18.3%) had symptomatic esophageal stricture, 12 (10.0%) had aspiration pneumonia of grade 2, and 7 (5.8%) had mediastinal emphysema of grade 2. Comparison of the 22 subjects with stricture with the 98 subjects without stricture showed significant differences in the rate of resection of >75% of the esophageal circumference, rate of whole circumference resection, and the required time for resection. The tumor size and the size of the resected tissue sample also differed between the 2 groups. The groups did not differ in age, sex, alcohol intake, and smoking; location, macroscopic, and histological tumor findings; chest pain; or use of anticoagulants for comorbidities. In multivariate analysis, tumor size and whole circumference resection were independent risk factors for stricture. Furthermore, in 45 subjects with resection of >75% of the esophageal circumference, whole resection of the esophagus was the only independent risk factor for stricture.

This study suggests that ESD has a strong therapeutic effect on superficial esophageal neoplasms; however, a greater extent of resection of the esophagus increases the risk of postoperative esophageal stricture. Preventive measures against development of postoperative stricture require further study.

## INTRODUCTION

Esophageal carcinoma is the eighth most common cancer worldwide, but incidences vary greatly among regions, with the highest rates found in Asia, southern and eastern Africa, and northern France. The annual mortality of esophageal carcinoma is close to 100 per 100,000 and also varies widely among countries.^1,2^ There are 2 main types of esophageal carcinoma: squamous cell carcinoma and adenocarcinoma. The morbidity of squamous cell carcinoma is greater in the Chinese, Korean, Japanese, and African populations, whereas that of adenocarcinoma is greater in the Anglo-Celtic population.^3^ In Japan, esophageal carcinoma is most frequent in men in their 60s and the male-to-female ratio is around 6:1.^4^ More than 90% of cases of esophageal carcinoma are squamous cell carcinoma, and the most common site is the middle thoracic esophagus (Mt).^4^

Treatment for esophageal neoplasms includes endoscopic resection, radiotherapy, chemotherapy, and surgical resection. In cases with a depth of invasion of epithelium or lamina propria mucosae, almost no metastasis occurs and endoscopic therapy is recommended.^5–7^ If the lesion reaches the muscularis mucosae to the upper submucosa (≤200 μm), about 20% of cases have metastases,^8,9^ and this is also an indication for endoscopic therapy. In contrast, patients with esophageal neoplasms with other than these depths of invasion are not suitable for endoscopic therapy.

Endoscopic mucosal resection (EMR) for gastrointestinal neoplasms has been conducted for many years, but en bloc resection has only been applied in cases with a tumor of less than about 2 cm. Ono^10^ and Yamamoto et al^11^ first described cases of early gastric cancer treated with endoscopic submucosal dissection (ESD). Subsequently, ESD has been performed in cases requiring wider resection and those with large superficial spreading gastric cancer. Oyama et al^12^ described ESD using a hook knife for early esophageal carcinoma, and various ESD techniques have been developed to achieve en bloc resection for circumferential or subcircumferential large superficial spreading esophageal neoplasms.^13^

The esophageal location is anatomically close to the lungs and heart, and complications may lead to serious conditions. An upper gastrointestinal endoscopy with a long procedure time may induce aspiration pneumonia. Also, the esophagus wall is thin and mediastinal emphysema may occur after ESD, with Oyama et al^12^ finding this complication in 6% of 102 patients with esophageal carcinoma undergoing ESD. Ono et al^14^ found an incidence of esophageal stricture of 18% after ESD for 107 lesions in 84 patients with esophageal carcinoma, with stricture occurring in only 6 of 74 patients (8.1%) who underwent <75% resection of the esophageal circumference, but in 9 of 10 patients (90%) who underwent >75% resection. Circumference resection and histological depth are reliable risk factors for stricture after ESD,^15,16^ but a detailed study of all risk factors for stricture after ESD has not been fully performed.^17^

Esophageal stricture after ESD can be treated by endoscopic balloon dilation, but several dilation procedures are needed, which prolongs hospitalization and compromises quality of life (QOL). Local injection of steroids may be effective in patients with esophageal stricture due to corrosive esophagitis,^18^ and steroid therapy is useful for treatment of stricture after ESD.^19^ However, the effects and risks of steroid therapy are not fully verified. The current retrospective study was performed to evaluate the efficacy of ESD, complications such as esophageal stricture after ESD, and the efficacy of local injection of steroids for stricture in patients with superficial esophageal neoplasms.

## SUBJECTS AND METHODS

The study was performed in 120 consecutive patients with superficial esophageal neoplasms who underwent ESD from January 2009 to December 2013, including patients with several lesions and those undergoing fractional excision, and excluding patients who took oral steroids. Superficial esophageal neoplasms included squamous cell carcinoma, tubular adenocarcinoma, high-grade intraepithelial neoplasia, and other cancer. The ESD procedure was performed according to previous reports.^12^ Of the 120 patients, 45 underwent >75% resection of the esophageal circumference by ESD. In these 45 patients, 8 patients who underwent ESD before November 2010 and 2 patients who underwent ESD in April and July 2013 did not receive local injection of steroids. The other 35 patients who underwent ESD from December 2010 received local injection of steroids, which was performed based on the report by Hashimoto et al.^19^ Steroids were locally injected 3 times under endoscopy immediately, 1 week and 2 weeks after ESD, and 0.2 mL of triamcinolone (50 mg/5 mL) was injected into the margins and floor of ulcers that developed in several locations after ESD.

**FIGURE 1:**
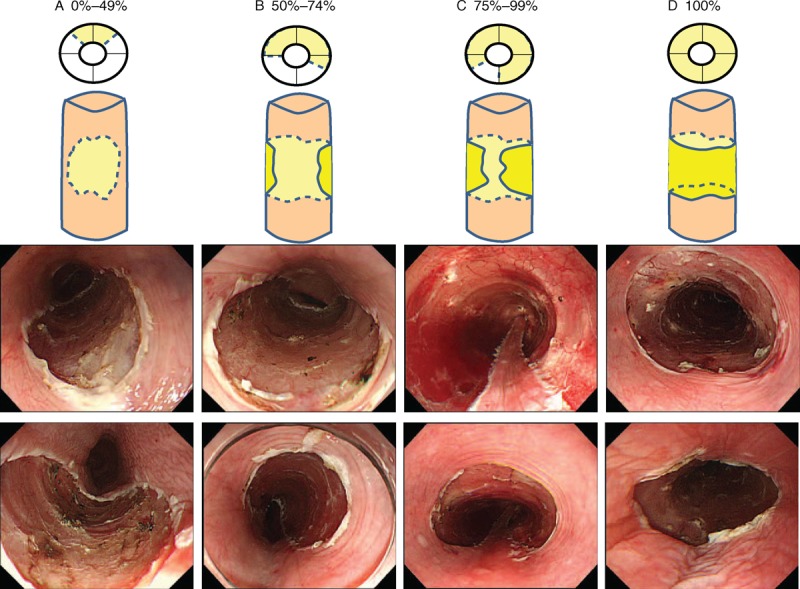
Illustrative images and endoscopic views showing grading of mucosal defects after ESD in the esophagus, based on the extent of resection of the esophageal circumference. (A) <50%. (B) 50% to <75%. (C) 75% to less than whole. (D) Whole circumference. ESD = endoscopic submucosal dissection.

The effect of ESD, the severity of esophageal stricture after ESD, and the efficacy and safety of local injection of steroids were retrospectively studied to identify preventive measures for stricture. En bloc resection was defined as single-piece resection of the tissue confirmed by endoscopic examination. Complete en bloc resection was defined as resected tissue that was pathologically negative for abnormal cells in surgical margins. The area of resection was classified according to the definitions by Katada et al^20^ of 25%, 50%, 75%, and whole resection of the esophageal circumference (Figure 1). The depth of tumor invasion was defined as follows^14,19,21^: EP, invasion of epithelium; LPM, invasion through the basement membrane to the lamina propria mucosa; MM, invasion to the muscularis mucosa; SM1, submucosal invasion (≤200 μm below the muscularis mucosa into the submucosa); and SM2, submucosal invasion (>200 μm into the submucosa).

Mediastinal emphysema was diagnosed based on x-ray or abdominal computed tomography (CT) images. Adverse events were graded according to the National Cancer Institute Common Terminology Criteria for Adverse Events, version 4.0 (CTCAEv 4.0 URL: http://ctep.cancer.gov/protocolDevelopment/electronic_applications/ctc.htm#ctc_40). This study was approved by the ethical review board of Kagoshima University Graduate School of Medical and Dental Sciences.

Statistical analysis was performed using SPSS software (SPSS Inc, Chicago, IL). The area under the receiver operating characteristic curve was used to determine cutoff values for further analysis. Multivariate analysis was performed using logistic regression analysis. Comparisons between 2 groups were performed by Mann–Whitney *U* test, χ^2^ test, or Fisher exact test, as appropriate. *P* < 0.05 was considered to be significant.

## RESULTS

### Characteristics of Subjects

The mean age of the subjects was 70.2 years; there were 110 men and 10 women, and 31 subjects had multiple lesions (Table 1). A total of 165 lesions were removed by ESD. The most common site of esophageal neoplasms was the Mt, which was observed in 94 subjects, and the most common macroscopic type was IIc in 101 lesions. The most common histological type was squamous cell carcinoma in 129 lesions.

**TABLE 1:**
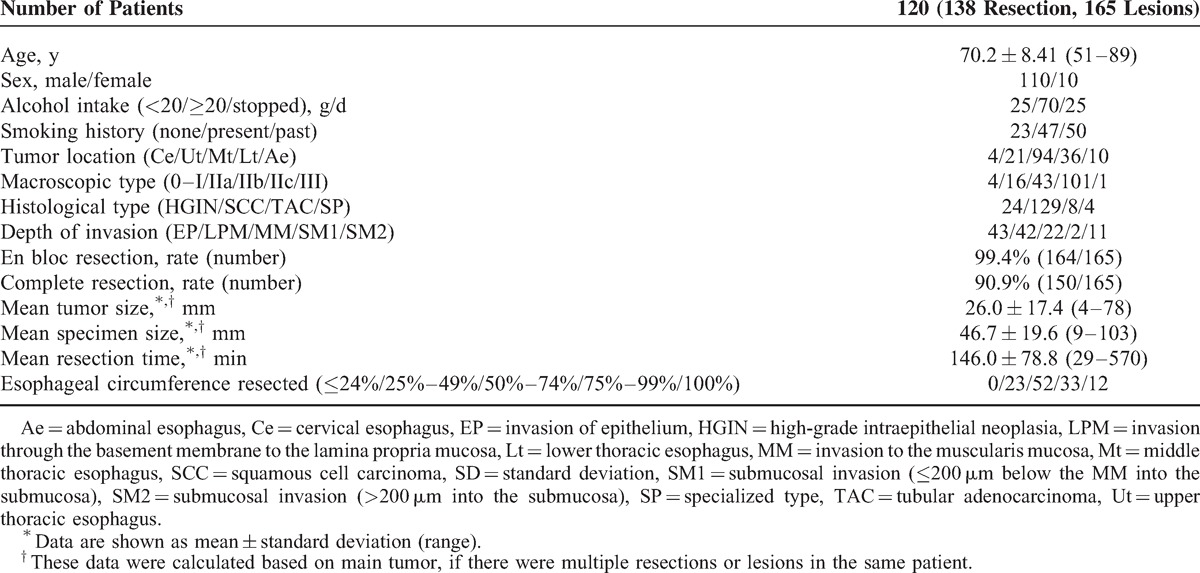
Clinical Characteristics of Patients

### Results for ESD of the Esophagus

En bloc resection and complete en bloc resection were performed for 164 (99.4%) and 150 (90.9%) of the 165 lesions, respectively (Table 1). The mean long tumor diameter was 26.0 mm, the mean long diameter of 120 resected tissue samples including the main tumor was 46.7 mm, and the mean operating time in the 120 procedures for the main tumor was 146.0 minutes.

### Adverse Events in ESD of the Esophagus

Postoperative adverse events included symptomatic esophageal stricture (esophageal stenosis, grade 2) in 18.3% of cases, aspiration pneumonia (pneumonitis, grade 2) in 10.0%, and mediastinal emphysema (mediastinal disorder, grade 2) in 5.8%. Endoscopic perforation, secondary hemorrhage, or events requiring surgical procedures were not observed (Table 2).

**TABLE 2:**
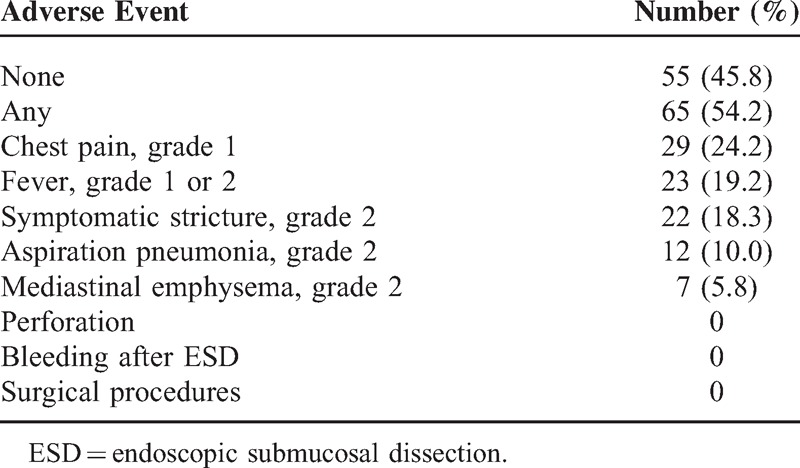
Adverse Events After ESD

### Comparison Between Stricture and Nonstricture Groups

**FIGURE 2:**
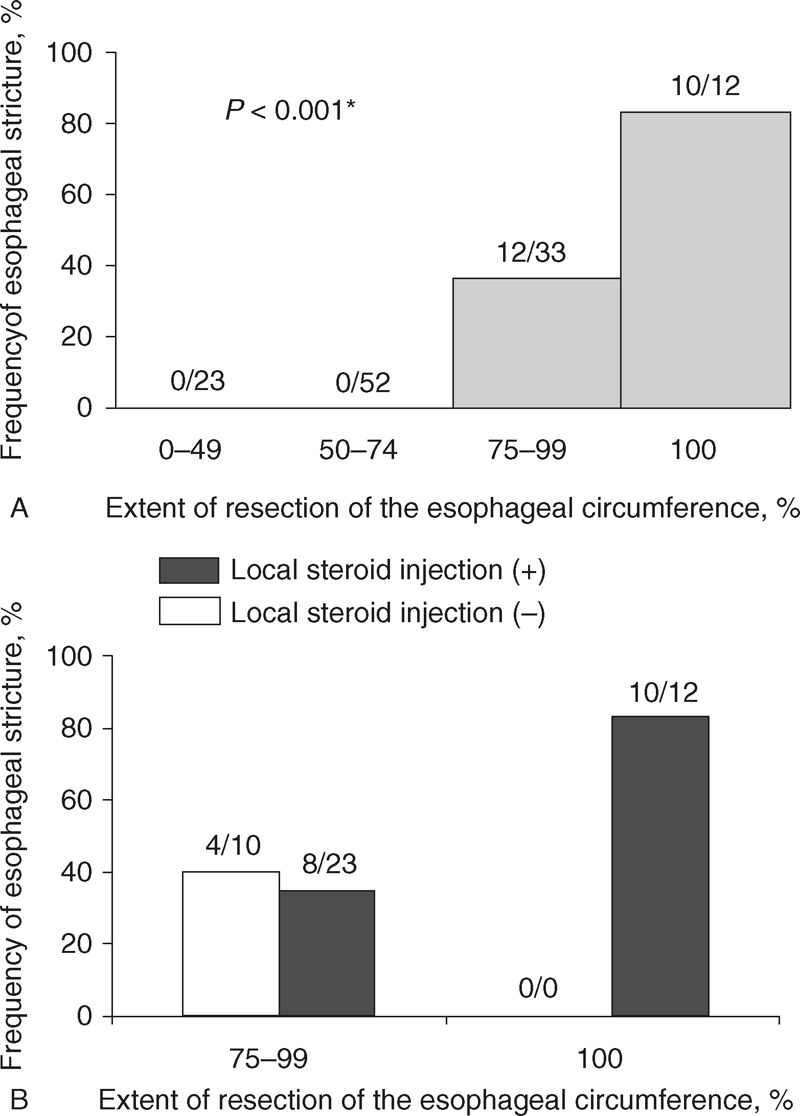
Frequency of symptomatic esophageal stricture formation after ESD, based on the extent of resection of the esophageal circumference. (A) All patients (n = 120). (B) Subjects who underwent resection of >75% of the esophageal circumference (n = 45), with or without local steroid injection. ^∗^Chi-square test. ESD = endoscopic submucosal dissection.

The background characteristics of 22 subjects who developed esophageal stricture after ESD and 98 subjects without esophageal stricture are compared in Table 3. There were no significant differences in age, sex, alcohol intake, and smoking; or in tumor location, macroscopic type, histological type, and depth of invasion between the 2 groups. The long diameters of the lesion (tumor size) and the resected tissue sample (specimen size) were significantly greater in the stricture group (*P* < 0.001), and the time required for tumor resection (resection time) was significantly longer in the stricture group (*P* < 0.01). The percentage of subjects undergoing resection of >75% or the whole esophageal circumference was also significantly greater in the stricture group (each *P* < 0.001). In addition, as the resection area increased, the incidence of postoperative esophageal stricture became greater (Figure 2A, *P* < 0.001). Endoscopic findings of fibrosis, chest pain during ESD, and fever >38°C after ESD, heparin administration instead of an anticoagulant for comorbidity during ESD, and use of anticoagulants for treatment of comorbidity after ESD did not differ between the groups.

**TABLE 3:**
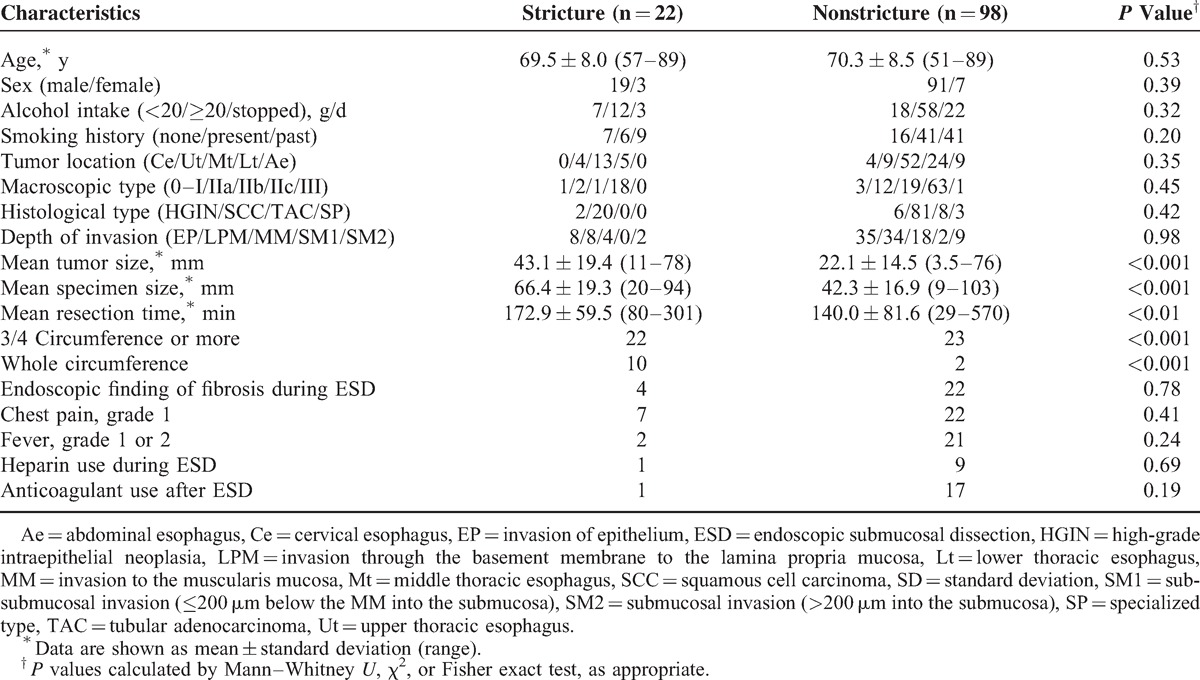
Comparison Between Cases With and Without Stricture in all Patients

In multivariate analysis using tumor size, specimen size, resection time, and >75% resection, there were no independent risk factors for postoperative stricture. In contrast, in a similar multivariate analysis in which whole circumference resection was used instead of >75% resection, in addition to tumor size, specimen size, and resection time, the tumor size and whole circumference resection were independent risk factors for stricture (Table 4). The circumference of the resected area was also closely associated with the tumor size, specimen size, and resection time. Therefore, multivariate analysis was performed using age, sex, and whole circumference resection, and whole circumference resection emerged as an independent risk factor for stricture (relative risk 41.47, 95% confidence interval [CI] 7.94–215.39, *P* < 0.001). Replacement of whole circumference resection with >75% resection in this analysis resulted in no independent risk factors.

**TABLE 4:**
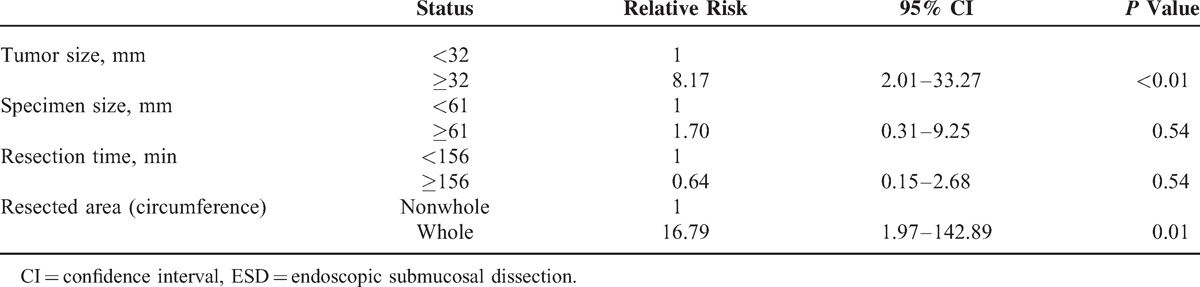
Multivariate Analysis of Factors Associated With Stricture After ESD

Univariate analysis limited to the 45 subjects who underwent resection of >75% of the esophageal circumference showed significant differences in the frequency of whole circumference resection and chest pain in the stricture and nonstricture groups (Table 5). In contrast, there were no differences in tumor size, specimen size, and resection time. The rate of local steroid injection in patients with stricture was similar to that in patients without stricture among a subgroup of patients whose esophageal circumference resection was 75% or more (Table 5). The percentage of stricture in patients treated with local steroid injection among a restricted group including 23 patients with 75% to 99% esophageal circumference resection was 34.8%, and this was similar to that in patients without local steroid injection (Figure 2B). In multivariate analysis using age, sex, whole circumference resection, and chest pain, whole circumference resection was the only independent risk factor for stricture (relative risk 7.83, 95% CI 1.30–47.27, *P* = 0.03). A bivariate analysis using whole circumference resection and chest pain also gave similar results (relative risk of whole circumference resection 7.00, 95% CI 1.18–41.54, *P* = 0.03).

**TABLE 5:**
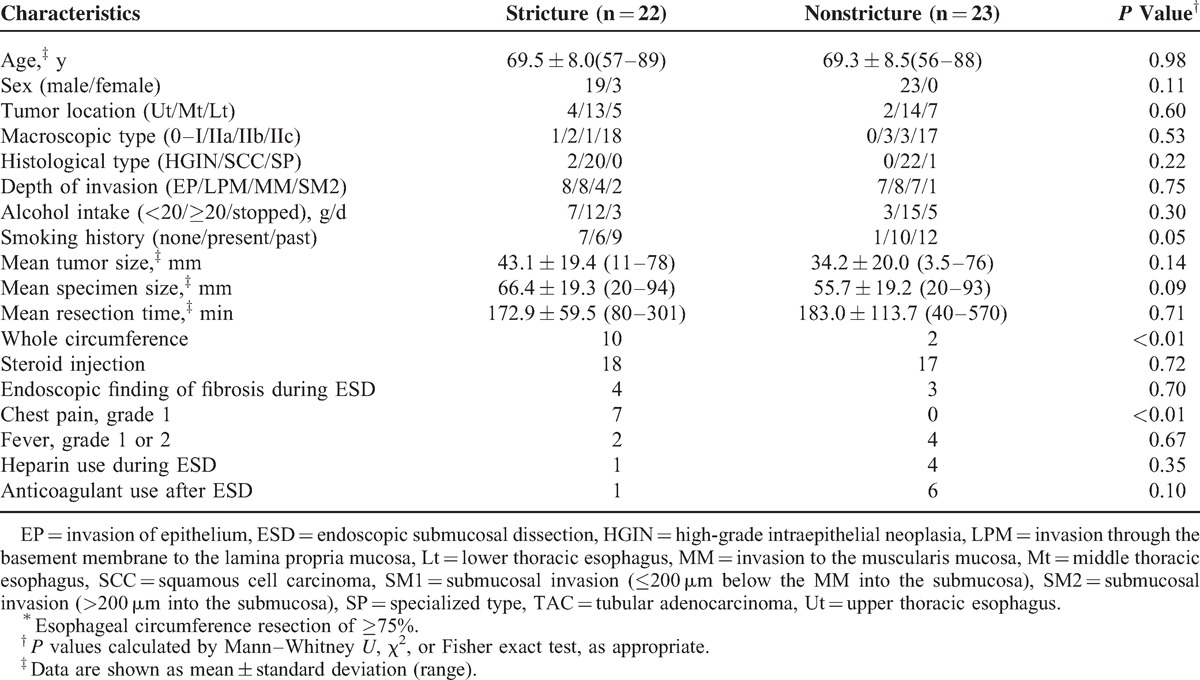
Comparison Between Cases With and Without Stricture in a Subgroup of Patients^∗^

## DISCUSSION

This study evaluated the effect and complications of ESD in 120 patients with superficial esophageal neoplasms. As in previous reports,^5,12,22–25^ the rates of en bloc resection of 99.4% (164/165) and complete en bloc resection of 90.9% (150/165) were high, and there were no fatal complications in our study. This suggests that ESD is an effective treatment for superficial esophageal neoplasms. However, postoperative esophageal stricture is a complication that affects QOL and was observed in 18% of the patients in this study. Resection of >75% of the esophageal circumference was a risk factor for stricture, and whole circumference resection was an independent risk factor in all patients and in a subgroup with >75% resection. Local injection of steroids has also been found to be useful for treatment of stricture,^19,26,27^ but we found no effect of local steroid injection on stricture.

Lewis et al^28^ performed EMR in 73 cases of Barrett esophageal carcinoma and identified >50% resection of the esophageal circumference as an independent risk factor for esophageal stricture. The reported incidence of stricture after ESD in patients with superficial esophageal carcinoma ranges from 5.0% to 17.2%,^23–25^ and the resected area is a risk factor. However, there are few studies of risk factors for esophageal stricture after ESD, and relationships between potential risk factors and stricture are difficult to evaluate in meta-analysis.^17^

Shi et al^16^ recently suggested that circumference resection of >75% was an independent risk factor for stricture. In the current study, the extent of esophageal circumference resection by ESD was associated with stricture, but only whole circumference resection was an independent risk factor. Shi et al enrolled more patients compared with our study, but 82.6% of the patients underwent ESD with a circumference range of <50%. In addition, 94.1% of patients with a resected circumference of >75% had postoperative stricture. In contrast, approximately half of the patients undergoing >75% resection of the esophageal circumference did not develop postoperative stricture in our study. The reason for the difference in the frequency of stricture between the 2 studies is unclear, but these results suggest that other factors may also contribute to postoperative stricture. In light of these findings, subjects in our study who underwent >75% resection of the esophageal circumference were divided into stricture and nonstricture groups to compare background factors. In multivariate analysis, the only independent risk factor was whole circumferential resection. We note that the depth of invasion was not associated with stricture in our study, whereas Shi et al^16^ found this to be an independent risk factor. However, Shi et al did not report the number of cases with whole circumferential resection. Collectively, these results suggest that patients with esophageal neoplasms who undergo resection of >75% of the esophageal circumference or whole circumferential resection are at high risk of stricture after ESD. However, the incidence of stricture may vary depending on the study population; host factors, such as single-nucleotide polymorphisms; and environmental factors, such as dietary habit.

Mediastinal emphysema was also observed as a complication after ESD, with incidences similar to those in a previous report.^23,24^ In contrast, the incidence of aspiration pneumonia in this study was 10%, which was greater than those reported by Takahashi et al^24^ (mean age 67 years) and Higuchi et al^25^ (median age 68 years). This might be because the subjects in our study were older (mean 70.2, median 71.0), and the number of smokers was greater (81%) than in the previous studies. Unclear diagnostic criteria for pneumonia may also have influenced the results, and a multicenter cohort study is needed after diagnostic criteria are established.

In patients undergoing >75% resection of the esophageal circumference, Hashimoto et al^19^ compared 21 cases with local injection of triamcinolone for stricture prevention with a control group of 20 cases without this procedure. Triamcinolone was found to be effective for stricture prevention and decreasing the mean number of balloon dilation procedures. Yamaguchi et al^29^ also divided patients undergoing >75% resection of the esophageal circumference into 2 groups of 19 cases administered oral prednisolone and 22 patients who underwent preventive balloon dilation. The incidence of postoperative esophageal stricture and the number of patients requiring therapeutic balloon dilation were lower in the oral prednisolone group.^29^ In our study, 35 of the 45 subjects undergoing >75% resection of the esophageal circumference received local injection of steroids for stricture prevention, but this procedure was not related to development of stricture. This finding suggests that the preventive effect of local injection of steroids on development of stricture is limited, and a larger study should be performed to identify patients who are indicated for local injection of steroids. The effect of oral intake of steroids should also be evaluated in a larger number of patients.

There are several limitations in this study. First, the design was retrospective. A previous prospective study of ESD in patients with esophageal carcinoma showed good efficacy,^23,25^ but the sample size was small. Thus, we believe that the current results are significant. Second, some of our subjects received local injection of steroids, which might have influenced the results. However, the effect of steroids is unclear and might be quite small. Within these limitations, we conclude that ESD has good efficacy in the treatment of superficial esophageal neoplasms. However, complications such as postoperative esophageal stricture are of concern. In particular, patients undergoing whole resection of the esophageal circumference are at high risk for esophageal stricture, and methods for prophylaxis of stricture are required.

In conclusion, ESD has a strong therapeutic effect on superficial esophageal neoplasms, but a greater extent of resection of the esophagus increases the risk of postoperative esophageal stricture, and whole circumference resections were independent risk factors for stricture. There is currently no reliable treatment to prevent development of postoperative stricture after ESD, and preventive measures against postoperative stricture require further study.

## References

[1] Herszényi L Tulassay Z Epidemiology of gastrointestinal and liver tumors. *Eur Rev Med Pharmacol Sci* 2010; 14:249–258. 20496531

[2] Holmes RS Vaughan TL Epidemiology and pathogenesis of esophageal cancer. *Semin Radiat Oncol* 2007; 17:2–9. 17185192 10.1016/j.semradonc.2006.09.003

[3] Hongo M Nagasaki Y Shoji T Epidemiology of esophageal cancer: Orient to Occident. Effects of chronology, geography and ethnicity. *J Gastroenterol Hepatol* 2009; 24:729–735. 19646015 10.1111/j.1440-1746.2009.05824.x

[4] Tachimori Y Ozawa S Fujishiro M Comprehensive registry of esophageal cancer in Japan, 2006. *Esophagus* 2014; 11:21–47. 10.1007/s10388-016-0531-y PMC4824839 27110229

[5] Fujishiro M Yahagi N Kakushima N Endoscopic submucosal dissection of esophageal squamous cell neoplasms. *Clin Gastroenterol Hepatol* 2006; 4:688–694. 16713746 10.1016/j.cgh.2006.03.024

[6] Shimizu Y Tsukagoshi H Fujita M Long-term outcome after endoscopic mucosal resection in patients with esophageal squamous cell carcinoma invading the muscularis mucosae or deeper. *Gastrointest Endosc* 2002; 56:387–390. 12196777 10.1016/s0016-5107(02)70043-6

[7] Katada C Muto M Momma K Clinical outcome after endoscopic mucosal resection for esophageal squamous cell carcinoma invading the muscularis mucosae—a multicenter retrospective cohort study. *Endoscopy* 2007; 39:779–783. 17703385 10.1055/s-2007-966761

[8] Ancona E Rampado S Cassaro M Prediction of lymph node status in superficial esophageal carcinoma. *Ann Surg Oncol* 2008; 15:3278–3288. 18726651 10.1245/s10434-008-0065-1

[9] Oyama T Miyata Y Shimatani S Lymph node metastasis of m3 and sm1 esophageal cancer [in Japanese]. *I Cho (Stomach Intestine)* 2002; 37:71–74.

[10] Ono H Endoscopic submucosal dissection for early gastric cancer. *Chin J Dig Dis* 2005; 6:119–121. 16045601 10.1111/j.1443-9573.2005.00206.x

[11] Yamamoto Y Fujisaki J Hirasawa T Therapeutic outcomes of endoscopic submucosal dissection of undifferentiated-type intramucosal gastric cancer without ulceration and preoperatively diagnosed as 20 millimetres or less in diameter. *Dig Endosc* 2010; 22:112–118. 20447204 10.1111/j.1443-1661.2010.00945.x

[12] Oyama T Tomori A Hotta K Endoscopic submucosal dissection of early esophageal cancer. *Clin Gastroenterol Hepatol* 2005; 3 (7 suppl 1):S67–70. 16013002 10.1016/s1542-3565(05)00291-0

[13] Oyama T Counter traction makes endoscopic submucosal dissection easier. *Clin Endosc* 2012; 45:375–378. 23251884 10.5946/ce.2012.45.4.375 PMC3521938

[14] Ono S Fujishiro M Niimi K Long-term outcomes of endoscopic submucosal dissection for superficial esophageal squamous cell neoplasms. *Gastrointest Endosc* 2009; 70:860–866. 19577748 10.1016/j.gie.2009.04.044

[15] Ono S Fujishiro M Niimi K Predictors of postoperative stricture after esophageal endoscopic submucosal dissection for superficial squamous cell neoplasms. *Endoscopy* 2009; 41:661–665. 19565442 10.1055/s-0029-1214867

[16] Shi Q Ju H Yao LQ Risk factors for postoperative stricture after endoscopic submucosal dissection for superficial esophageal carcinoma. *Endoscopy* 2014; 46:640–644. 24830402 10.1055/s-0034-1365648

[17] Sun F Yuan P Chen T Efficacy and complication of endoscopic submucosal dissection for superficial esophageal carcinoma: a systematic review and meta-analysis. *J Cardiothorac Surg* 2014; 9:78. 24885614 10.1186/1749-8090-9-78 PMC4052291

[18] Holder TM Ashcraft KW Leape L The treatment of patients with esophageal strictures by local steroid injections. *J Pediatr Surg* 1969; 4:646–653. 5371094 10.1016/0022-3468(69)90492-8

[19] Hashimoto S Kobayashi M Takeuchi M The efficacy of endoscopic triamcinolone injection for the prevention of esophageal stricture after endoscopic submucosal dissection. *Gastrointest Endosc* 2011; 74:1389–1393. 22136782 10.1016/j.gie.2011.07.070

[20] Katada C Muto M Manabe T Esophageal stenosis after endoscopic mucosal resection of superficial esophageal lesions. *Gastrointest Endosc* 2003; 57:165–169. 12556777 10.1067/mge.2003.73

[21] Kuwano H Nishinuma Y Ohtsu A Guidelines for diagnosis and treatment of carcinoma of the esophagus. April 2007 edition: Part I. Edited by the Japan Esophageal Society. *Esophagus* 2008; 5:61–73.

[22] Ishihara R Iishi H Uedo N Comparison of EMR and endoscopic submucosal dissection for en bloc resection of early esophageal cancers in Japan. *Gastrointest Endosc* 2008; 68:1066–1072. 18620345 10.1016/j.gie.2008.03.1114

[23] Repici A Hassan C Carlino A Endoscopic submucosal dissection in patients with early esophageal squamous cell carcinoma: results from a prospective Western series. *Gastrointest Endosc* 2010; 71:715–721. 20363414 10.1016/j.gie.2009.11.020

[24] Takahashi H Arimura Y Masao H Endoscopic submucosal dissection is superior to conventional endoscopic resection as a curative treatment for early squamous cell carcinoma of the esophagus (with video). *Gastrointest Endosc* 2010; 72:255–264. 20541198 10.1016/j.gie.2010.02.040

[25] Higuchi K Tanabe S Azuma M A phase II study of endoscopic submucosal dissection for superficial esophageal neoplasms (KDOG 0901). *Gastrointest Endosc* 2013; 78:704–710. 23680178 10.1016/j.gie.2013.04.182

[26] Isomoto H Yamaguchi N Minami H Management of complications associated with endoscopic submucosal dissection/endoscopic mucosal resection for esophageal cancer. *Dig Endosc* 2013; 25 suppl 1:29–38. 23368404 10.1111/j.1443-1661.2012.01388.x

[27] Hanaoka N Ishihara R Takeuchi Y Intralesional steroid injection to prevent stricture after endoscopic submucosal dissection for esophageal cancer: a controlled prospective study. *Endoscopy* 2012; 44:1007–1011. 22930171 10.1055/s-0032-1310107

[28] Lewis JJ Rubenstein JH Singal AG Factors associated with esophageal stricture formation after endoscopic mucosal resection for neoplastic Barrett's esophagus. *Gastrointest Endosc* 2011; 74:753–760. 21820109 10.1016/j.gie.2011.05.031 PMC3481547

[29] Yamaguchi N Isomoto H Nakayama T Usefulness of oral prednisolone in the treatment of esophageal stricture after endoscopic submucosal dissection for superficial esophageal squamous cell carcinoma. *Gastrointest Endosc* 2011; 73:1115–1121. 21492854 10.1016/j.gie.2011.02.005

